# Metabolomics after trauma in experimental models- a systematic review

**DOI:** 10.1007/s00423-025-03917-z

**Published:** 2026-01-10

**Authors:** Galo Stückelberger, Matthias Weuster, Anisa Hana, Christian Hübner, Yannik Kalbas, Hans-Christoph Pape, Felix Karl-Ludwig Klingebiel, Roman Pfeifer

**Affiliations:** 1 Department of Trauma and Orthopedics Surgery, Cantonal Hospital Winterthur, Brauerstrasse 15, 8401 Winterthur, Switzerland; 2 Department of Surgical Research, Harald Tscherne Laboratory for Orthopaedic and Trauma Research, Zurich University Hospital, Zurich, Switzerland; 3 Clinic for Trauma Surgery, Diako Hospital Flensburg, Flensburg, Germany; 4 Institute of Intensive Care Medicine, University Hospital of Zurich, Zurich, Switzerland; 5 Department of Trauma Surgery, University Hospital Zurich, University of Zurich, Ramistrasse 100, 8091 Zurich, Switzerland

**Keywords:** Metabolomics, Hemorrhagic shock, Trauma, Animal models, Resuscitation, Glutamine, Succinate, Redox balance, Purines, Plasma therapy

## Abstract

**Background:**

Hemorrhagic shock (HS) is a major risk factor for mortality and complications after severe trauma. Yet, complex mechanisms on a cellular and physiological level are still not fully understood, Metabolomics offers a powerful tool to unravel these complex biochemical alterations following HS and trauma (T). However, no systematic synthesis of metabolomic findings in in standardized translational animal models of HS and HS + T exists to date.

**Objectives:**

To systematically review and compare metabolomic alterations in animal models of isolated hemorrhagic shock and hemorrhagic shock with trauma, highlighting key metabolic pathways and their modulation by resuscitation strategies.

**Methods:**

We performed a systematic review on Pubmed and EMBASE to identify relevant metabolomic changes measured in with mass spectrometry in translational animal models following HS and HS + T. Studies were categorized into two groups in regard to our main objective: Isolated HS and HS + T. Key metabolite changes were extracted and analyzed qualitatively across seven major metabolic domains: energy, amino acids, purines, arginine/urea cycle, lipids, sulfur/creatine, and redox balance.

**Results:**

Overall, 25 studies were included in our analysis. HS and HS + T both exhibited consistent changes in lactate (↑), succinate (↑), glutamine (↑), and acylcarnitines (↑), indicating hypoxia-driven glycolysis, mitochondrial dysfunction, and enhanced proteolysis. HS + T models displayed more pronounced alterations in branched-chain amino acids, purine catabolites (urate, inosine), and markers of oxidative stress. ATP depletion and glutathione imbalance were common, particularly in the trauma group. Plasma resuscitation partially corrected several abnormalities, especially in redox, purine, and glutamine pathways, compared to saline.

**Conclusions:**

Metabolic responses to hemorrhagic shock are reproducible across animal models and are amplified by the presence of trauma. Trauma accentuates proteolysis, oxidative stress, and nucleotide turnover. Plasma-based resuscitation appears superior to crystalloid in correcting these derangements. These findings support the exploration of targeted metabolic therapies and advocate for metabolomics-informed resuscitation strategies in trauma care.

**Supplementary Information:**

The online version contains supplementary material available at 10.1007/s00423-025-03917-z.

## Introduction

Trauma is a leading cause of death and disability worldwide, particularly among young adults, with over 6 million trauma-related deaths occurring annually. A significant proportion of these deaths are due to uncontrolled hemorrhage, often within the first hours post-injury, making it the most preventable cause of trauma-related mortality [1]. Despite advances in prehospital care and damage control strategies, mortality and morbidity remain high, partly due to the complex and poorly understood pathophysiological changes on a cellular level that occur during hemorrhagic shock and trauma.

Hemorrhagic shock initiates a cascade of metabolic disturbances, including acidosis, coagulopathy, and hypothermia—the so-called “lethal triad” [2]. Severe trauma additionally induces systemic metabolic dysregulation characterized by catabolism, insulin resistance, and altered amino acid utilization. This constellation of metabolic responses, often referred to as “traumatic diabetes,” defines a specific post-injury phenotype or “metabotype” associated with poor outcomes [3, 4].

Metabolomics—the large-scale study of small-molecule metabolites—has emerged as a powerful tool to characterize these dynamic biochemical changes. Technologies such as NMR, LC-MCS,spectroscopy, and ultra-performance LC–MS (UPLC-MS) have enabled the detection of trauma-induced alterations in energy metabolism, amino acid catabolism, purine turnover, lipid oxidation, and redox balance in animal models of HS [5–7]. These techniques offer insight into the biochemical signature of shock, providing potential biomarkers and therapeutic targets.

While numerous animal studies have examined metabolic changes in either isolated hemorrhagic shock or combined trauma and hemorrhage, no systematic review to date has compared these models side by side. Distinguishing between metabolic alterations caused by hemorrhage alone versus those amplified by additional trauma is crucial for translational research and precision resuscitation strategies.

This systematic review aims to compare metabolomic signatures betweenNMRE animal models of isolated hemorrhagic shock (HS) and HS combined with trauma (HS + T), and to evaluate the influence of different resuscitation strategies. By synthesizing the available evidence across key metabolic pathways—including glycolysis, the tricarboxylic acid (TCA) cycle, amino acid and purine metabolism, lipid oxidation, and redox homeostasis—this work seeks to identify core pathophysiologic patterns and highlight areas for future investigation.

Our hypothesis is that the metaboloc reaction to hemorrhagic shock and the combination with musculoskeletal trauma results in a different metabolic pattern.

## Methods

The reporting of this systematic review adheres to the Preferred Reporting Items for Systematic Reviews and Meta-Analyses (PRISMA) guidelines (http://www.prisma-statement.org/).

### Study design and scope

This study was designed as a systematic review of metabolomic studies conducted in animal models of HS and HS + T. The review aimed to synthesize reported metabolic changes, categorize them by biological pathway, and compare patterns between isolated HS and HS + T conditions.

### Literature search strategy

A systematic literature search was performed using PubMed and Embase including publications from 2000–2023 on 03.01.2023. The combination of controlled vocabulary and regular search terms was employed, in conjunction with MESH/EMTREE terms. Additional sources were identified either outside of the systematic search strategy as literature of other publications or were recommended by experts in the field. An overview of the search terms is provided in the supplementary content. Search results were screened manually by two independent reviewers. Titles and abstracts were reviewed for relevance, followed by full-text evaluation based on predefined inclusion criteria.

### Inclusion and exclusion criteria

Studies were included if they met the following criteria:

- Conducted in animal models (rats, pigs, primates)
- Induced hemorrhagic shock, either alone or in combination with trauma
- Employed metabolomic analysis techniques (NMR, LC–MS, UPLC-MS)
- Reported quantitative or qualitative metabolic changes in plasma, serum, or tissue
- Published as original article
- Written in English

Studies were excluded if they:

- Lacked of reporting relevant information
- Were of low scientific quality

### Study categorization

Eligible studies were categorized into two groups:

- Group 1: Isolated Hemorrhagic Shock (HS)
- Studies using hemorrhagic shock models without additional traumatic injuries
- Group 2: Hemorrhagic Shock and Trauma (HS + T)
- Studies incorporating trauma components (e.g., liver crush, lung contusion, fracture, laparotomy, or brain injury) in addition to hemorrhage

### Data extraction and synthesis

Data were independently extracted from each study and cross-validated by two reviewers. The following variables were recorded:

- First author and publication year
- Animal species and strain
- Model description (type of trauma or HS, resuscitation strategy)
- Sampling time points
- Metabolomic platform used (e.g., NMR, LC–MS, UPLC-MS)
- Key altered metabolites and their reported direction (increase/decrease)
- Affected metabolic pathways (e.g., glycolysis, TCA cycle, amino acid metabolism, lipid metabolism, purine metabolism, redox balance)

The results were stratified according to biological pathway domains, based on prior frameworks from comparable metabolomic studies in trauma research [1, 5, 6]. No meta-analytic statistics were applied due to heterogeneity in study design, metabolomic techniques, and outcome reporting.

### Data analysis

All included studies were reviewed in detail, and reported metabolites were extracted and tabulated. Each identified metabolite was assigned to one of seven predefined metabolic domains: (1) energy metabolism, (2) amino acid metabolism, (3) purine metabolism, (4) arginine and urea cycle, (5) lipid metabolism, (6) sulfur- and creatine-related metabolism, and (7) redox regulation. The categorization of metabolites into these pathways was based on established biochemical databases and prior metabolomic trauma research [1, 2], including KEGG, HMDB, and the framework published by D’Alessandro et al. [3].

To ensure comparability, we grouped metabolites by both experimental group (Isolated Hemorrhagic Shock [HS] vs. Hemorrhagic Shock + Trauma [HS + T]) and sampling timepoint, typically described in the original studies as:

- **Early phase** (0–60 min post-shock or trauma),
- **Resuscitation phase** (immediately after fluid or blood resuscitation),
- **Late/recovery phase** (> 2–6 h post-resuscitation).
- If studies did not specify exact timepoints, they were assigned to the nearest matching category based on described experimental protocols.

A metabolite was considered “relevant” if it fulfilled the following criteria:

- It was explicitly mentioned in the results or discussion section as being altered in response to HS or HS + T.
- It showed a reported directional change (increase or decrease) compared to sham or baseline.
- It was detected in ≥ 50% of studies within a group, or was emphasized mechanistically by the study authors.

Quantitative synthesis was conducted by calculating the frequency of metabolite reporting per group. To normalize across groups of different study numbers (HS: *n* = 9; HS + T: *n* = 16), we expressed frequencies as percentages of total studies per group.

In addition, whenever possible, we documented the temporal phase in which each metabolite alteration occurred (early, resuscitation, late). This allowed us to not only identify consistent metabolic pathways but also to infer potential time-dependent shifts in metabolic priorities during trauma and recovery.

Discrepancies in metabolite assignment or classification were resolved by consensus discussion among the reviewers.

## Results

**Fig. 1:**
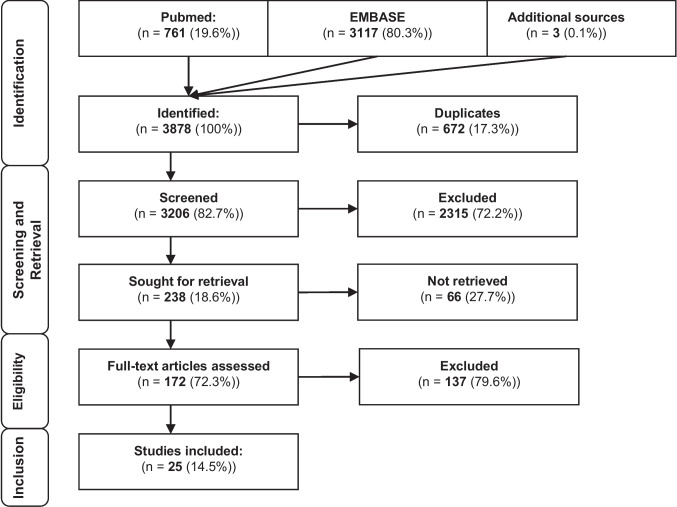
Note: Flowchart of the sytematic review

A total of 25 studies were included in this systematic review after the screening process (Fig. 1), comprising 9 models of isolated HS and 16 models combining HS + T (Tables 1, 2). These experimental studies were conducted in rats and pigs, employing a variety of metabolomic platforms including LC–MS, UPLC-MS, and NMR spectroscopy. The key metabolomic alterations were organized into seven core metabolic domains, which are Energy Metabolism, Amino Acid Metabolism and Glutaminolysis, Purine Catabolism and Salvage Pathways, Arginine Metabolism and the Urea Cycle, Lipid Metabolism and β-Oxidation, Sulfur and Creatine Metabolism, Redox Homeostasis.

**Table 1:** Included studies for isolated hemorrhagic shock

Author	Animal	Model	Time points	Techniques
Bogren et al. [8]	Rats	Ischemia–reperfusion	Pre-HS, 3 h post-reperfusion	LC–MS
D'Alessandro et al. [1]	Rats	HS	0, 10, 20, 30 min	UPLC-MS
D'Alessandro et al. [9]	Rats	HS + resuscitation	0, 10, 20, 30 min + resuscitation	UPLC-MS
Duan et al. [10]	Rats	HS (intestinal focus)	Basal, post-shock, 4 h post	LC–MS + 16S rRNA
Lusczek et al. [11]	Pigs	HS + hypothermia	Basal, 45 min shock, 1/4/8 h resusc	NMR
Mulier et al. [12]	Pigs	HS + resuscitation	Basal, shock, hourly for 20 h	NMR
Reisz et al. [13]	Rats	HS	0, 15, 60 min	UPLC-MS + isotope tracing
Reisz et al. [14]	Rats, pigs, primates, humans	HS (comparative)	Basal, 90 min post-shock	LC–MS + isotope standards
Scribner et al. [15]	Pigs	HS	Basal, 45 min shock, 8/23/48 h post	NMR (^1^H & ^31^P)

**Table 2:** Included studies for hemorrhagic shock combined with trauma

Author	Animal	Model	Time points	Techniques
D’Alessandro et al. [1]	Rats	Trauma vs HS	Baseline, 5–35 min post-HS	UPLC-MS
Daniels et al. [16]	Swine	HS + femoral fractures + resuscitation	Baseline, during, post-resusc., 2 h	LC–MS
Determan et al. [17]	Pigs	HS + lung and liver injury	Post-surgery, 45 m HS, 2–20 h resusc	NMR
Carrasco Laserna et al. [18]	Pigs	HS + femoral fracture + hypothermia	Baseline, 30 m, post-HS	LC–MS
Lexcen et al. [19]	Pigs	HS + lung and liver injury	Baseline, 45 m, 8 h resuscitation	NMR
Li et al. [20]	Rats	HS + lung, liver, laparotomy	Baseline, 3 h, 5 h post-HS	NMR
Lusczek et al. [21]	Pigs	HS + lung, liver, laparotomy	Baseline, 45 m, 3/9/21/48 h	NMR
Lusczek et al. [22]	Pigs	HS + lung, liver, laparotomy	Baseline, 45 m, multiple up to 48 h	NMR
Shen et al. [23]	Rats	Brain injury + fracture	Baseline, injury, 21 days	LC–MS
Shi et al. [24]	Rats	HS (spleen and thymus)	Baseline, post-injury	LC–MS
Slaughter et al. [25]	Rats	HS + multiorgan analysis	Baseline, 30–45 m	LC–MS
Slaughter et al. [5]	Rats	HS + small bowel trauma	Baseline, 45 m, 3 h post-resuscitation	LC–MS
Wang et al. [26]	Rats	HS + trauma	Baseline, 24 h post-injury	LC–MS
Witowski et al. [27]	Pigs	HS + lung, liver, laparotomy	Baseline, 45 m, 2–48 h	NMR
Witowski et al. [28]	Pigs	HS + lung, liver, laparotomy	Baseline, 45 m, 2 h	LC–MS
Xu et al. [29]	Rats	HS + tail amputation	Baseline, 1 h post-HS	LC–MS

### Energy metabolism

One of the most consistent findings across both HS and HS + T models was the elevation of lactate, a hallmark of anaerobic glycolysis and tissue hypoxia. This was observed in 15 of the 18 studies reporting lactate, reflecting the shift toward non-oxidative energy production following hemorrhagic shock. D’Alessandro et al. [1] and Witowski et al. [2] demonstrated that lactate levels significantly increased during the early post-shock phase, especially under crystalloid resuscitation.

Pyruvate levels were also found to be elevated, although with more variability—some studies reported transient increases, while others described a drop in pyruvate concentration during prolonged shock, suggesting a bottleneck in mitochondrial pyruvate entry [3]. This pattern was particularly evident in models with prolonged or delayed resuscitation [4].

Succinate, a central TCA cycle intermediate and a recognized marker of ischemia–reperfusion injury, was significantly elevated in both groups, with a stronger and more sustained increase in HS + T models. Slaughter et al. [5] reported that succinate accumulation correlated with the severity of mitochondrial dysfunction and the extent of reperfusion-induced oxidative stress.

In terms of cellular energy reserves, ATP levels were consistently decreased, particularly in models with prolonged hypoperfusion or inadequate resuscitation. Moore et al. [30] observed that plasma resuscitation helped to partially restore ATP concentrations compared to saline, suggesting improved mitochondrial recovery and substrate availability.

Together, these findings highlight a profound disruption of energy homeostasis in hemorrhagic shock, with trauma acting as a metabolic amplifier that further depletes cellular energy reserves.

### Amino acid metabolism and glutaminolysis

A broad spectrum of amino acids showed dynamic changes following HS and HS + T. Notably, glutamine levels increased markedly in both groups, particularly during the early hours after resuscitation. This suggests increased glutaminolysis, potentially compensating for impaired glucose oxidation and supporting the synthesis of glutathione and other redox-related molecules [1, 7]. Duan et al. [10] confirmed that intestinal glutamine metabolism plays a pivotal role in host defense and tissue repair in HS models.

Branched-chain amino acids (BCAAs)—specifically valine, leucine, and isoleucine—were also elevated, especially in HS + T models. Their consistent upregulation (observed in over 10 studies) reflects increased muscle proteolysis and peripheral tissue breakdown as a response to stress and inflammation. Shi et al. [4] reported that BCAA accumulation coincided with increased protease activity and elevated plasma cytokines.

Alanine, a key transamination product, was significantly increased in models of both isolated and combined trauma, supporting the hypothesis that amino acid interconversion is upregulated under shock conditions to fuel gluconeogenesis and ammonia detoxification [13].

Collectively, these findings demonstrate a catabolic shift in amino acid metabolism after hemorrhagic insult, which is more pronounced when trauma is present. These amino acids serve as both energy substrates and building blocks for stress adaptation and immune modulation.

### Purine catabolism and salvage pathways

Multiple studies reported profound changes in purine metabolism, particularly involving hypoxanthine, inosine, urate, and allantoin, all of which are downstream products of ATP degradation. These molecules were consistently elevated after shock and trauma, suggesting increased nucleotide turnover and oxidative stress.

Xu et al. [6] and Slaughter et al. [5] observed that urate levels rose sharply during shock and continued to rise after saline resuscitation, whereas plasma-treated animals had a more controlled response. This suggests that plasma resuscitation may facilitate purine salvage pathways, preserving nucleotide pools and reducing the burden of oxidative byproducts.

D’Alessandro et al. [1] demonstrated that plasma-based resuscitation promoted inosine and xanthine clearance, possibly by enhancing mitochondrial flux and reducing xanthine oxidase activity. These results underscore the link between purine metabolism and mitochondrial health during hemorrhagic shock.

### Arginine metabolism and the urea cycle

Arginine, a substrate for nitric oxide (NO) synthesis and the urea cycle, was largely unchanged or decreased post-shock, despite the broad increase in other amino acids. Downstream metabolites such as ornithine and citrulline were often elevated, especially in the HS + T group [7]. These shifts suggest a redirection of arginine metabolism toward urea cycle flux rather than NO production, potentially limiting vasodilatory and endothelial-protective effects.

Wang et al. [7] reported that the accumulation of polyamines such as spermidine and spermine in saline-treated animals reflects an arginase-driven metabolic response, associated with cellular stress and platelet dysfunction. This pathway appears attenuated in plasma-treated models, suggesting a resuscitation-dependent modulation of arginine flux.

### Lipid metabolism and β-oxidation

Lipid metabolism was consistently altered, with increases in acylcarnitines—notably acetylcarnitine, butyrylcarnitine, and propionylcarnitine—indicating enhanced β-oxidation. These changes reflect a metabolic switch toward fat utilization in the face of carbohydrate oxidation failure [31]. Carrasco Laserna et al. [3] observed that ketone bodies such as β-hydroxybutyrate and acetoacetate accumulated rapidly in both HS and HS + T models, but reached higher concentrations in the trauma group.

Interestingly, Witowski et al. [2] found that plasma resuscitation partially mitigated excessive acylcarnitine accumulation, suggesting better metabolic regulation and lipid handling compared to crystalloids. This supports the role of plasma not only in volume replacement but also in metabolic buffering.

### Sulfur and creatine metabolism

The sulfur-containing amino acids taurine and hypotaurine, known for their antioxidant and osmotic regulation roles, were significantly elevated after HS and trauma. Xu et al. [6] demonstrated that their accumulation correlated with increased oxidative stress markers and mitochondrial injury.

Creatine and phosphocreatine, critical for rapid ATP regeneration, also increased in most studies, especially during the post-resuscitation period. These changes likely represent a compensatory response to ATP depletion and disrupted mitochondrial energetics [32].

### Redox homeostasis

Markers of oxidative stress were consistently dysregulated across models. Glutathione (GSH) depletion, increased oxidized glutathione (GSSG), and elevated 5-oxoproline levels were noted particularly in saline-treated animals [1, 5, 14]. Succinate, a known activator of reactive oxygen species (ROS) during reperfusion, remained elevated in many models, especially in the HS + T group.

D’Alessandro et al. [1] and Reisz et al. [14] showed that plasma-based resuscitation attenuated these redox perturbations, supporting its role in preserving mitochondrial redox poise and minimizing reperfusion injury.

## Discussion

This systematic review provides a comprehensive synthesis of metabolomic studies in experimental animal models of hemorrhagic shock (HS), with and without additional trauma (HS + T). By grouping findings into key metabolic domains, it reveals both overlapping and divergent metabolic signatures between the two groups. Overall, trauma emerged as a metabolic amplifier, intensifying biochemical dysregulation observed in hemorrhagic shock alone.

### Energy crisis and glycolytic shift

A defining feature of hemorrhagic shock is the early and sustained switch from aerobic to anaerobic metabolism, driven by tissue hypoperfusion and mitochondrial dysfunction. The consistent elevation of lactate and pyruvate across models confirms activation of glycolysis, while the decrease in ATP and accumulation of succinate points to impaired oxidative phosphorylation and TCA cycle stalling [1–6].

This pattern was especially marked in HS + T models, likely reflecting prolonged ischemia, higher systemic inflammation, and greater metabolic demand due to tissue injury. Succinate, beyond being a TCA intermediate, also acts as a pro-inflammatory and pro-oxidant molecule upon reperfusion, contributing to secondary injury [5]. The relative normalization of these metabolites under plasma resuscitation, as seen in several studies [1, 2, 6], suggests a resuscitation fluid-dependent modulation of mitochondrial recovery and energy restoration.

### Trauma-driven proteolysis and amino acid remodeling

The increase in glutamine, alanine, and BCAAs (valine, leucine, isoleucine) reflects an enhanced proteolytic state under metabolic stress. These amino acids serve not only as energy substrates but also support immune responses, gluconeogenesis, and glutathione synthesis [1, 7, 13, 33]. Trauma intensified these effects: HS + T models consistently exhibited higher amino acid concentrations than HS alone, likely due to the added burden of muscle and tissue breakdown.

Glutamine, in particular, plays a dual role as a fuel for rapidly dividing cells (e.g., lymphocytes) and as a precursor for glutathione, contributing to redox balance. Experimental studies have shown that glutamine supplementation improves outcomes in shock by restoring ATP levels and protecting mitochondria [33]. The metabolomic data support this therapeutic rationale, as glutamine-related pathways were more preserved in plasma-resuscitated animals.

### Purine breakdown reflects cellular injury and oxidative load

The accumulation of hypoxanthine, inosine, urate, and allantoin across both HS and HS + T groups indicates accelerated ATP turnover and mitochondrial injury. The catabolism of purines, often triggered by ischemia and oxidative stress, produces ROS and inflammatory mediators [5, 6, 30]. HS + T models showed higher levels of these metabolites, reinforcing trauma’s role in exacerbating systemic oxidative injury.

Interestingly, plasma resuscitation was associated with lower purine catabolite concentrations and increased salvage pathway activity [1, 14], suggesting better preservation of nucleotide pools and reduced redox burden. This supports the growing view that resuscitation fluids influence not only hemodynamics but also intracellular metabolism.

### Arginine metabolism: a metabolic crossroad

Arginine is a key substrate for both nitric oxide (NO) synthesis and the urea cycle. Its stable or decreased levels post-shock, despite generalized amino acid elevation, suggest preferential consumption via NO or polyamine pathways [7, 28]. The rise in citrulline, ornithine, and polyamines (spermidine, spermine) in HS + T models—particularly under saline resuscitation—reflects a shift toward arginase activity, which may reduce NO bioavailability and contribute to vasoconstriction, inflammation, and endothelial dysfunction [5, 34].

These findings emphasize the delicate balance between protective and pathologic arginine utilization in trauma. Plasma resuscitation appears to limit polyamine overproduction, possibly preserving NO signaling and endothelial function [1, 14].

### Lipid metabolism: from energy supply to stress marker

The consistent elevation of acylcarnitines and ketone bodies suggests increased reliance on β-oxidation and lipid-derived energy sources during shock. These shifts likely represent adaptive responses to glucose shortage and mitochondrial dysfunction [3, 31]. However, sustained increases in acylcarnitines—particularly in HS + T—may signal incomplete oxidation and lipid overload, which can further impair mitochondrial function.

Plasma resuscitation appeared to better regulate these lipid changes than saline, perhaps by improving perfusion and reducing oxidative lipid peroxidation [2, 14]. These findings suggest a dual role for lipid metabolites: as energy substrates and as markers of mitochondrial distress.

### Sulfur metabolism and the creatine-phosphate system

Metabolites such as taurine, hypotaurine, creatine, and phosphocreatine were elevated in both groups, particularly under trauma conditions. These molecules are central to osmoregulation, cell membrane stabilization, and rapid energy buffering, all of which are crucial in the post-shock period [6, 32].

Taurine is also a potent antioxidant, and its accumulation may represent a compensatory mechanism against oxidative damage. The preservation of these sulfur pathways under plasma resuscitation suggests that such fluids may contribute not only to hemodynamic stability but also to cellular stress adaptation.

### Redox imbalance and resuscitation-dependent correction

Perhaps the most striking finding across models is the consistent oxidative stress signature: elevated succinate, depleted GSH, increased GSSG, and accumulation of 5-oxoproline [1, 5, 14]. These indicate mitochondrial damage, impaired redox buffering, and excessive ROS generation.

Trauma worsened these features, but plasma-based resuscitation partially restored redox homeostasis, possibly by replenishing antioxidant precursors and supporting mitochondrial function [1, 2, 14]. These data reinforce the concept that resuscitation fluids are not metabolically inert, and that their composition can significantly influence cellular recovery.

### Summary and translational relevance

**Fig. 2:**
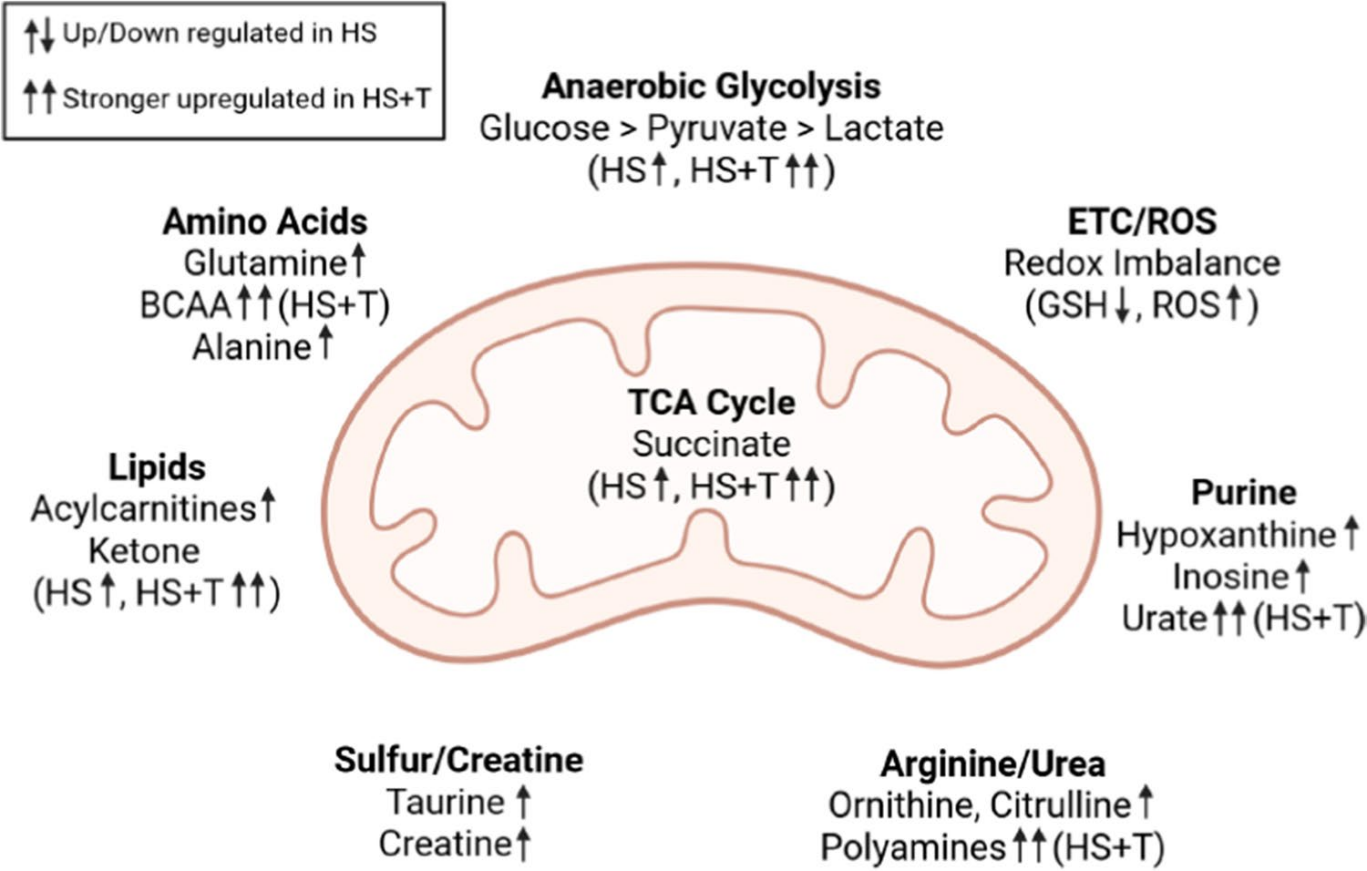
Metabolic alterations in hemorrhagic shock (HS) and combined hemorrhagic shock with trauma(HS + T), Created in BioRender. Klingebiel, F. (2025)

The convergence of findings across species, techniques, and models strengthens the case for core metabolic patterns in hemorrhagic shock. However, the presence of trauma consistently amplifies metabolic derangement, affecting nearly every pathway examined. These differences must be considered when extrapolating from isolated HS models to clinical trauma populations (Fig. 2).

Moreover, the type of resuscitation fluid emerged as a critical variable: plasma offered consistent advantages over saline in mitigating acidosis, preserving redox balance, and supporting key metabolic pathways. This supports ongoing efforts to implement plasma-first resuscitation strategies in the prehospital setting.

## Discussion

Based on the results of this systematic review, we conclude the following main findings:

1. In the early phase after hemorrhagic shock, a consistent increase in glycolytic metabolites such as lactate and pyruvate reflects a rapid switch to anaerobic energy production, indicating acute mitochondrial dysfunction and impaired oxidative phosphorylation.
2. Succinate accumulation occurred predominantly in the early post-shock and reperfusion phase and was more pronounced in combined trauma models, suggesting enhanced TCA cycle disruption and reactive oxygen species (ROS) production.
3. Amino acid catabolism, especially glutamine, BCAAs, and alanine, was activated across both groups, with greater magnitude in trauma models. These shifts are linked to proteolysis, immune activation, and gluconeogenic substrate provision.
4. Purine catabolites (e.g., hypoxanthine, inosine, urate) rose substantially during the resuscitation phase, particularly in HS + T, indicating ATP degradation, oxidative stress, and impaired nucleotide salvage pathways.
5. Redox imbalance was reflected by increased 5-oxoproline, depletion of glutathione, and prolonged oxidative signatures. Plasma-based resuscitation attenuated these changes, suggesting a metabolic regulatory effect of plasma beyond volume replacement.

In the early phase following hemorrhagic shock, lactate and pyruvate concentrations rise rapidly, signaling a shift to anaerobic glycolysis due to tissue hypoperfusion and mitochondrial inhibition. This glycolytic switch is a hallmark of metabolic stress and was consistently reported across both groups. Notably, HS + T models exhibited even higher levels of these metabolites, indicating intensified mitochondrial dysfunction as a result of combined injury and hypoxia [1, 3, 5]. Lactate is a well-established biomarker that reflects the physiological state of patients. Nevertheless, lactate is most frequently regarded by clinicians as an indicator of "ischemia", signifying the presence of bleeding or a systemic pathology. In order to facilitate a more comprehensive interpretation of lactate levels, it is imperative that the fundamental knowledge in this field is augmented. A frequently overlooked aspect in clinical decision-making is the potential for the reduction of lactate levels following resuscitation to be attributable not only to clinical improvement, but also, to a certain extent, to the dilution of body volume [35]. Consequently, the dynamics of clinical parameters and the interactions between laboratory values and administered drugs/transfusions must be taken into account.

Another consistent finding was the accumulation of succinate, a key TCA cycle intermediate, particularly evident during the reperfusion phase. Succinate accumulation promotes reverse electron transport at mitochondrial complex I, resulting in reactive oxygen species (ROS) generation and subsequent tissue injury. Although detected in both groups, succinate tended to reach higher levels in HS-only models, possibly due to earlier sampling timepoints in some trauma studies [2, 5]. Nonetheless, its presence highlights a shared ischemia–reperfusion phenotype across injury types. Nevertheless, succinate is not a parameter that is routinely measured – particularly in the context of acute situations, but rather more commonly in the context of chronic diseases or research.

Amino acid metabolism was also significantly altered in both HS and HS + T models. Increases in glutamine, branched-chain amino acids (BCAAs), and alanine were consistently observed during the resuscitation period. These shifts are indicative of proteolysis, immune cell activation, and increased substrate demand for gluconeogenesis. Trauma models showed greater magnitude of change, suggesting enhanced systemic catabolism and inflammation. Glutamine’s dual role—as a fuel for immune cells and as a glutathione precursor—also links amino acid metabolism to redox regulation [1, 3, 33]. The present findings may be of significance in the context of post-traumatic nutrition, and they have previously been investigated in a number of translational models. These findings imply a beneficial impact on the patient's metabolism [36]. The clinical data available to date appear to support the administration of branched-chain amino acids (BCAAs) to specific patient cohorts. However, it is evident that the evidence level requires further improvement to identify specific patient cohorts, the optimal timing of administration, dosages and potential contraindications [37–39].

Purine catabolism was another prominent metabolic feature, particularly during the recovery phase. Increased levels of hypoxanthine, inosine, and urate indicate ongoing ATP degradation and oxidative stress. These metabolites were more elevated in trauma models, consistent with more extensive tissue damage. Importantly, studies show that plasma-based resuscitation modulates this pathway, reducing purine catabolite accumulation and potentially preserving cellular energy stores [5, 30]. It is therefore conceivable that these markers could be utilised in decision-making processes, with the objective of determining the energy requirements of the individual.

Finally, redox imbalance was reflected in elevated levels of 5-oxoproline and reduced glutathione pools, pointing to impaired ROS detoxification and oxidative organ stress. This effect was more pronounced in HS + T models. However, plasma resuscitation was associated with partial restoration of glutathione balance and reduction of oxidative markers, suggesting that plasma exerts metabolic as well as hemodynamic effects in trauma care [1, 14]. This finding lends further support to the use of balanced transfusion strategies and early plasma use in cases of haemorrhagic shock, which are now the primary treatment methods [40]. Consequently, this strategy has the potential to not only support the physiology in terms of volume and electrolyte balance, but also in terms of oxidative stress and glutathione-repletion [41].

Taken together, these findings demonstrate that while the same core metabolic pathways are disrupted in both HS and HS + T, trauma amplifies the intensity and persistence of metabolic dysregulation. Moreover, resuscitation strategy—particularly the use of plasma—plays a decisive role in shaping the metabolic recovery. These insights have implications for translational research, biomarker development, and resuscitation strategies in clinical trauma care.

In addition to biological relevance, the clinical feasibility of metabolomic testing remains a limiting factor. Most analytical platforms used in experimental studies—including LC–MS, UPLC-MS, and high-resolution NMR—are not currently available as point-of-care tests (POCT). These technologies require centralized laboratory infrastructure, skilled personnel, and are associated with considerable cost and turnaround time. However, the successful implementation of lactate as a POCT parameter illustrates that selected metabolites can be effectively translated into rapid, bedside diagnostics. The long-term goal of trauma-focused metabolomics research should therefore be to identify robust and clinically meaningful metabolic markers that can be adapted for POCT use, enabling early risk stratification and more individualized resuscitation strategies.

## Conclusion

This systematic review demonstrates that hemorrhagic shock, both in isolation and in combination with trauma, induces profound and reproducible metabolic disturbances in animal models. Across 25 preclinical studies, consistent alterations were observed in energy metabolism, amino acid catabolism, purine turnover, lipid oxidation, redox balance, and mitochondrial function.

While many metabolic signatures were shared between isolated HS and HS + T models, the presence of trauma amplified the magnitude and duration of these disturbances—particularly in pathways associated with proteolysis, oxidative stress, and purine degradation. This suggests that trauma synergistically exacerbates the metabolic consequences of hemorrhage, highlighting the importance of studying both entities together in translational models.

Notably, several studies demonstrated that the choice of resuscitation fluid significantly modulates the metabolic response. Plasma-based resuscitation consistently outperformed crystalloid solutions in restoring redox balance, supporting mitochondrial function, and attenuating ATP and amino acid depletion. These findings reinforce the emerging concept that metabolic recovery is not solely a function of volume replacement, but also of metabolic reprogramming guided by the composition of resuscitation fluids.

Together, these insights provide a valuable foundation for future experimental and clinical research aimed at:

- Identifying metabolic biomarkers to guide trauma care and prognostication;
- Designing metabolically targeted resuscitation strategies, including glutamine- or antioxidant-based adjuncts;
- Refining animal models to better reflect the metabolic complexity of human trauma.

By deepening our understanding of trauma-induced metabolic dysregulation, metabolomics may ultimately help shape more precise, physiology-based interventions for hemorrhagic shock and trauma patients.

## Limitations

This review has several limitations that must be acknowledged. First, the included studies exhibited considerable heterogeneity in experimental design, including differences in animal species (e.g., rats vs. pigs), trauma type (e.g., liver crush, femoral fracture, lung contusion), and severity of hemorrhagic shock. These variations complicate direct comparisons and limit the generalizability of quantitative findings.

Second, the timing of metabolite sampling varied widely between studies, ranging from immediate post-shock to several hours after resuscitation. This temporal heterogeneity may mask dynamic metabolic shifts and make it challenging to draw conclusions about specific phases of shock and recovery.

Third, while the categorization of metabolites into functional pathways was based on recognized biochemical databases (e.g., KEGG, HMDB), the assignment of individual metabolites to discrete pathways is not always exclusive, and overlapping functions may introduce classification bias.

Fourth, many studies relied on different analytical platforms (e.g., LC–MS vs. NMR), which differ in sensitivity and coverage. As a result, some metabolites may have gone undetected in certain models, introducing reporting bias in our frequency-based summary.

Finally, and most importantly, this review is based entirely on preclinical animal studies, which raises concerns regarding the translatability of findings to human trauma physiology. Although animal models provide valuable mechanistic insights, they cannot fully replicate the complexity, comorbidities, and interindividual variability of human trauma patients.

Despite these limitations, the consistency of metabolic alterations across models reinforces the robustness of the core pathways identified and provides a solid foundation for translational research.

The current literature indicates that many metabolic responses observed in experimental trauma models are analogous to those seen in humans [42]. However, the degree of transferability is strongly influenced by the animal species used. Larger animals, such as pigs or sheep, tend to exhibit hemodynamic and metabolic responses that more closely resemble those of humans, whereas the majority of studies in this review were conducted in rodent models [43].

While our review provides important mechanistic insights, the clinical translation of metabolomic findings remains a major challenge. Future validation in human trauma cohorts is necessary to assess whether these metabolic patterns and pathway perturbations are conserved. Moreover, the clinical relevance of individual metabolites must be evaluated in the context of patient heterogeneity, co-existing conditions, and complex therapeutic environments.

## Supplementary Information

Below is the link to the electronic supplementary material.

Supplementary file1 (PDF 190 KB)

## Data Availability

No datasets were generated or analysed during the current study.

## Abbreviations

^1^H NMR: Proton Nuclear Magnetic Resonance
16S rRNA: 16S Ribosomal RNA Gene Sequencing
^31^P NMR: Phosphorus Nuclear Magnetic Resonance
Femoral fracture: Break in the femur
HMDB: Human Metabolome Database
HS: Hemorrhagic Shock
HS + Trauma: Hemorrhagic Shock and Trauma
Hypothermia: Induced Hypothermia
I/R: Ischemia and Reperfusion
Isotope standards: Internal Isotopically Labeled Standards
Isotope tracing: Stable Isotope Tracing
KEGG: Kyoto Encyclopedia of Genes and Genomes
Laparotomy: Surgical Opening of the Abdominal Cavity
LC-MS: Liquid Chromatography – Mass Spectrometry
Liver crush injury: Hepatic trauma induced by compression
Lung contusion: Pulmonary Contusion
NMR: Nuclear Magnetic Resonance
Resuscitation: Fluid/Blood Resuscitation
Tail amputation: Tail resection in rodents
UPLC-MS: Ultra Performance Liquid Chromatography – Mass Spectrometry
